# Sudden gains in internet cognitive therapy for social anxiety disorder in routine clinical practice

**DOI:** 10.1016/j.invent.2024.100788

**Published:** 2024-11-12

**Authors:** See Heng Yim, David M. Clark, Paul M. Salkovskis, Graham R. Thew

**Affiliations:** a Oxford Institute of Clinical Psychology Training, University of Oxford, Warneford Hospital, OX3 7JX Oxford, UK; b Oxford Centre for Anxiety Disorders and Trauma (OxCADAT), Department of Experimental Psychology, University of Oxford, The Old Rectory, Paradise Square, Oxford OX1 1TW, UK; c Oxford Health NHS Foundation Trust, Oxford, UK; d Department of Experimental Psychology, University of Oxford, UK

**Keywords:** Social anxiety, Internet interventions, Sudden gains, Effectiveness study, Cognitive behavioural therapy, IAPT

## Abstract

**Background:**

Sudden gains are large symptom improvements between consecutive therapy sessions. They have been shown to occur in randomised controlled trials of internet-delivered psychological interventions, but little is known about their occurrence when such treatments are delivered in routine clinical practice.

**Objective:**

This study examined the occurrence of sudden gains in a therapist-guided internet-delivered Cognitive Therapy intervention for social anxiety disorder (iCT-SAD) delivered in the UK NHS talking therapies for anxiety and depression (formerly known as IAPT services). It aimed to assess whether sudden gains were associated with better therapy outcomes, and examine changes in process variables around the period of sudden gains.

**Methods:**

The study examined sudden gains based on the Liebowitz Social Anxiety Scale. Of 193 treated patients, 146 provided sufficient data to permit analysis. Linear mixed effects models were used to examine the impact of sudden gains on clinical outcomes, and examine changes in negative social cognitions, self-focused attention, and depressed mood.

**Results:**

Seventy sudden gains were found among 57 participants. The occurrence rate of sudden gains was 39 %. Individuals who experienced sudden gains had a larger reduction in social anxiety symptoms at end of intervention and at three-month follow-up. There was evidence of a reduction in the frequency of negative social cognitions prior to the gain, whereas changes in self-focused attention occurred simultaneously with the gain. Depressed mood did not show significant changes over these timepoints.

**Conclusions:**

Approximately 2 in 5 patients experienced a sudden gain whilst accessing the iCT-SAD intervention in routine practice. They were associated with better clinical outcomes following treatment compared to those who did not experience a sudden gain.

## Highlights

• Sudden gains in social anxiety were found using iCT-SAD in routine practice
• Having a sudden gain was associated with a greater reduction in social anxiety at end of intervention and at follow-up
• Significant reductions in the frequency of negative cognitions occurred immediately prior to sudden gains

## 1 Introduction

Social anxiety disorder (SAD) is a common mental health condition that is persistent if untreated (Bruce et al., 2005). In the UK, the National Institute for Health and Care Excellence (NICE) guidelines recommend individual cognitive behavioural therapy (CBT) specific to SAD as a first-line treatment for adults with SAD (NICE, 2013). One of the two treatment protocols recommended is based on the Clark and Wells (1995) model of SAD. This cognitive therapy protocol (CT-SAD) is typically delivered in face-to-face sessions, but a new internet-delivered format of this protocol has strong empirical support from pilot studies and randomised controlled trials (RCT) in the UK (Stott et al., 2013; Clark et al., 2022) and Hong Kong (Thew et al., 2019, Thew et al., 2022). Internet interventions offer a cost-effective and more accessible alternative to face-to-face therapies that may overcome barriers of seeking and accessing therapy (Donker et al., 2015). As a result, clinical services such as those in the NHS Talking Therapies for anxiety and depression (NHS TTad) (formerly IAPT) programme have been keen to adopt internet interventions due to their potential benefits for clients such as flexibility in when and where they work on their treatment and the ability to work at their own pace (Thew, 2020). iCT-SAD has recently been implemented and evaluated across a group of NHS TTad services (Clark et al., in preparation).

Research on internet interventions for SAD shows that they are effective but much less is known about individual patient-level data (Kampmann et al., 2016). One approach to exploring individual patient trajectories is by examining sudden gains in therapy, where a large improvement in symptoms is observed between two consecutive therapy sessions. Tang and DeRubeis (1999) developed three criteria to identify sudden gains: the gain should be (1) large in absolute magnitude, (2) large relative to the previous symptom score, and (3) large relative to symptom fluctuation. Sudden gains (SGs) have been found to occur in in-person and online psychological therapies in a range of clinical populations including social anxiety (Bohn et al., 2013), OCD (Aderka et al., 2012) and depression (Kelly et al., 2005). Shalom and Aderka (2020) conducted a systematic review and meta-analysis on 50 studies that examined SGs across depression and different anxiety disorders. They found that on average, 34.65 % of participants experienced a SG, and the effect size on the primary outcome was significantly greater for individuals who experienced SGs.

However, out of the 50 included studies in the meta-analysis (Shalom and Aderka, 2020), only three examined social anxiety disorder (Bohn et al., 2013; Hofmann et al., 2006; Thorisdottir et al., 2018). Together with a more recent publication (Bisby et al., 2022), these all used data drawn from RCTs conducted in research settings. One might argue that RCTs might be more likely to find sudden gains given that the therapists may have received specialist training in the intervention, or that the participants would need to meet specific inclusion criteria. Moreover, most of the studies on SGs included modest sample sizes. For example, there were 45 participants across two treatment conditions for Thorisdottir et al. (2018) and 67 participants for Bohn et al. (2013). None of the included studies examined internet-based treatments for this population. More recently, Thew et al. (2023) explored sudden gains using data from the Clark et al. (2022) RCT, which compared face-to-face CT-SAD to iCT-SAD. They found that sudden gains were observed in 64 % of the participants in CT-SAD, and in 51 % of those in iCT-SAD. It is important to further the understanding of mechanisms of change in internet interventions for SAD and help identify potential targets that may require more therapist support for optimising the delivery of internet interventions.

### 1.1 Aims

This study aimed to examine sudden gains (SGs) in iCT-SAD. It aimed to replicate the approach of Thew et al. (2023) and extend it to a routine clinical setting. This study sought to understand whether differences in the frequency or nature of SGs might be observed when iCT-SAD is delivered as part of routine service provision.

First, we identified the frequency, magnitude of SG, and the likelihood of reversal of SGs. The primary aim was to examine whether experiencing a SG was associated with better social anxiety outcomes. The secondary aim was to examine how key process variables (negative social cognitions, self-focused attention and depressed mood) change around the period when sudden gains are found. Specifically, building on the previous study findings (Thew et al., 2023), we hypothesised that reductions in negative social cognition and self-focused attention would precede sudden gains but mood changes would not necessarily do so.

## 2 Method

### 2.1 Participants

The data were drawn from a larger study implementing iCT-SAD in NHS Talking Therapies for anxiety and depression (NHS TTad) (formerly IAPT) services (*N* = 193). Ethical approval was granted by the North East Tyne & Wear South Research Ethics Committee (REC 20/NE/0241). Specifically, we included people with a primary presenting problem of SAD who were willing and able to use an internet intervention. People had to be able to speak, read and write in English and if taking medication, agreed to continue their current dose during the course of the study. The exclusion criteria were people with marked clinical risk based on the service's assessment, and people who were concurrently on another clinical research study. Recruitment took place between August 2020 and January 2022 from six NHS TTad services across London and the Southeast. More detailed information about the implementation study is given in Clark et al. (in prep).

For the present study, participants with fewer than eight LSAS datapoints (*N* = 47) were not included in the analysis. This ensured that all analysed participants received a sufficient dose of therapy and provided an adequate amount and distribution of LSAS datapoints to allow the calculation of SGs.

### 2.2 Intervention

iCT-SAD is an online Cognitive Therapy intervention that replicates the treatment procedures from the traditional in-person Cognitive Therapy protocol (Clark et al., 2006). It is delivered via a secure online platform supported by a weekly telephone call with a trained therapist, along with brief written messages and one webcam call in the second week of treatment. Individuals can message their therapists in the messaging section on the platform. The modules include an individualised psychological formulation based on the Clark and Wells (1995) model, patient testimonies, multi-media materials, case examples, interactive activities where users are encouraged to complete a range of exercises, tasks and behavioural experiments. Treatment begins with eight core modules, typically completed over the initial two weeks. These are ‘Introducing the treatment’, ‘Getting started’, ‘Feeling self-conscious’, ‘Safety behaviours’, ‘My attention and safety behaviour experiment’, ‘Watching your conversation videos’, ‘Getting out of your head and into the world’, and ‘Behavioural experiments’. A range of further problem-specific modules are then available in order to tailor the treatment to the clients specific feared concerns. For more details of the design and development of the intervention, please refer to Stott et al. (2013) and Clark et al. (2022). It is typically delivered across 14 weeks, followed by up to 3 monthly booster sessions. For the present study, this timeline was followed appropriate but incorporated flexibility based on clinical need.

### 2.3 Therapists

A total of 33 therapists from the participating NHS TTad services provided the treatment. All were Psychological Wellbeing Practitioners (PWP) or High Intensity CBT therapists who had received specialist training in iCT-SAD and regular supervision from four experienced iCT-SAD practitioners.

### 2.4 Outcome measures

Outcome measures were completed weekly on the iCT-SAD website.

#### 2.4.1 Social anxiety measures

The self-report version of the 24-item Liebowitz Social Anxiety Scale (LSAS) (Baker et al., 2002) was used weekly.

#### 2.4.2 Process measures

##### 2.4.2.1 Negative social cognitions

The Social Cognitions Questionnaire (SCQ) (Clark, 2005) was measured weekly. This 22-item scale measures the frequency (range 1–5) and belief (range 0–100) ratings for a range of negative automatic cognitions in social anxiety.

##### 2.4.2.2 Self-focused attention

Two items on self-focused attention in social situations were taken from the Social Phobia Weekly Summary Scale measured weekly (SPWSS) (range 0–8) and the scale has been shown to have high internal consistency (Clark, 2005). One of the items asked about self-focused attention in general situations, and the other item asked about difficult social situations.

#### 2.4.3 Mood measures

As part of the routine outcome collection in NHS TTad services, the Patient Health Questionnaire-Depression Scale (PHQ-9) (Kroenke et al., 2001) for monitoring low mood weekly. A score above 9 indicates clinical ‘caseness’. Scores 5–9 indicates mild depression, 10–14 indicates moderate depression, 15–19 moderate-severe depression, and 20–27 indicates severe depression.

### 2.5 Data analysis

Analyses were performed in R Studio (R Core Team, 2021). The ‘suddengains’ R package (Wiedemann et al., 2020) was used to identify SGs. The ‘nlme’ R package (Pinheiro et al., 2018) was used for linear mixed-effects models.

#### 2.5.1 Identification of SGs

As iCT-SAD allows individuals to access the platform and work on their treatment at any time, the notion of discrete treatment ‘sessions’ is not applicable. Instead, we refer to treatment ‘week’. The week prior to the SG is referred to as week ‘n’ (pre-gain), and the week after the SG as ‘n+1’ (post-gain). SG identification was based on the self-report LSAS using the criteria outlined by Tang et al. (2005). For criterion one, we used a cut-off of 12 LSAS points, consistent with Thew et al. (2023) which was calculated based on the Reliable Change Index (RCI) (Jacobson and Truax, 1991) for the scale. Criterion two requires the gain to represent a drop of at least 25 % of the pre-gain score. Criterion three refers to the fact that the mean difference between the three pre- and post-gain measurements was >2.78 times the pooled standard deviations of these sessions' LSAS scores. Reversal of SG was defined as a loss of 50 % of the gain at any subsequent point of the intervention, for example, if the LSAS score drops from 60 to 40 between sessions (i.e. sudden gain), the gain is said to be reversed if a score of 50 or more is noted at any subsequent week. Where a participant experienced more than one SG, the gain with the larger magnitude was used as it was thought to be more clinically significant. If the magnitudes were the same, then the first SG would be used for the analysis.

#### 2.5.2 Hypothesis-driven analyses

Linear mixed-effects models with maximum likelihood estimation were used to (i) examine if the change in pre- to post-intervention LSAS scores significantly differed between participants with or without SGs, and (ii) explore changes in the frequency of, and degree of belief in, negative social cognitions (measured by SCQ), self-focused attention in general and difficult social situations (SPWSS), and depressed mood (PHQ) around the period of SGs. For (i), LSAS score was the dependent variable and the categorical fixed factors were time (post-intervention and three-month follow-up) as well as sudden gain status (present or absent). Participant was specified as a random effect. Baseline LSAS score was included as a covariate. For (ii), Timepoint around the SG (n-2, n-1, n, n+1, n+2, n+3) was specified as a categorical fixed factor, and participant as a random factor to allow for between-participant variation. The effect size measure used was Cohen's *d*. Visual inspection of the QQ plots showed normally distributed residuals for all models. Bonferroni correction was used due to multiple comparisons being tested.

#### 2.5.3 Missing data

Where participants had a missing LSAS score for a particular session, imputation was not performed since this can carry the risk of artificially influencing the calculation of sudden gains (see Tang et al., 2007). Instead, the ‘suddengains’ R package automatically makes adjustments for small amounts of missing data when calculating SGs (Wiedemann et al., 2020), setting a more stringent threshold for criterion three based on the values given in Lutz et al. (2013).

## 3 Results

### 3.1 Participant characteristics

The mean and median number of weeks the 146 participants were involved in the intervention was 14.53 (SD = 3.33) and 14 (range = 8–30).

Participants' demographic characteristics are listed in Table 1.

**Table 1:** Participants' demographics.

Variable		Value
Gender (n = 146)	Female	70
	Male	60
	Non-binary or prefer not to say	2
	N/A	14
Age (n = 135)	Mean	28.33 (SD = 7.76),
	Range	18-56
Ethnicity (n = 146)	White (British, Irish, any other)	103
	Mixed-Race	15
	Indian	1
	Chinese	1
	Any other Asian	2
	Caribbean	2
	African	1
	Any other Black	2
	Other	3
	Not stated	16

### 3.2 Characteristics of SGs in routine clinical practice

**Fig. 1:**
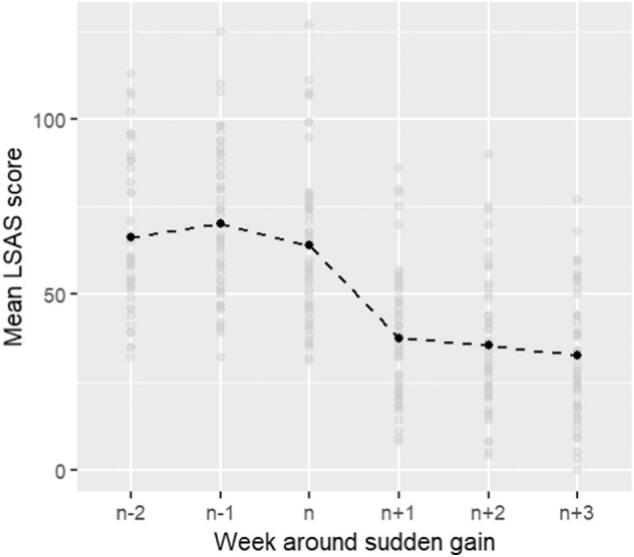
Mean LSAS scores around Sudden Gains.

**Fig. 2:**
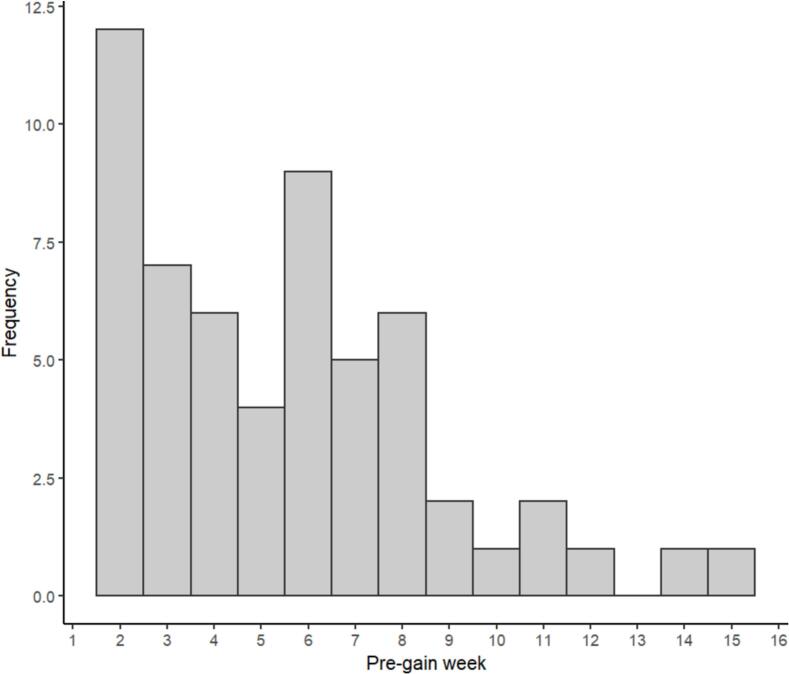
A Histogram showing the distribution pre-gain week (n) when SGs occurred.

Of the 146 participants, 57 experienced a SG (39 %). Forty-four participants had one SG, and thirteen had two SGs, giving a total of 70 SGs. There were three instances where SGs were not maintained (SG reversals, see Methods). The mean SG magnitude was 25.29 (*SD* = 11.27). Fig. 1 shows the mean LSAS scores around the period of SGs. Fig. 2 shows the distribution of pre-gain weeks when SGs occurred. SGs were distributed throughout the course of the intervention. The most common pre-gain weeks were two and six.

**Fig. 3:**
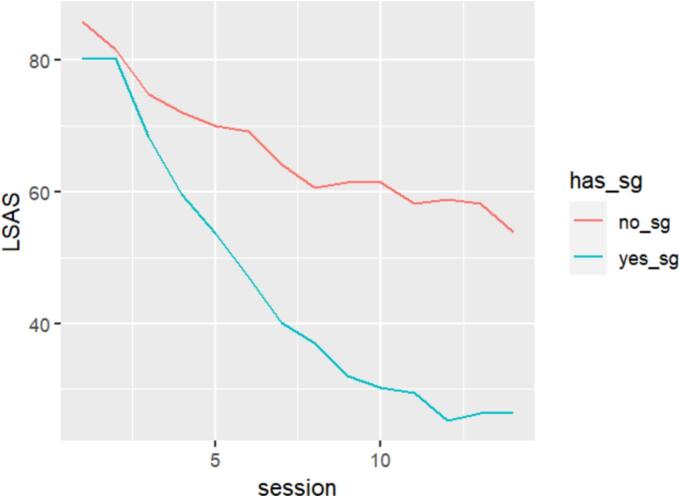
Symptom trajectories of the SG and non-SG groups.

To examine the possibility that SGs might simply be the result of longer treatment duration, the average number of weeks of treatment was calculated for the SG and no-SG groups. The average number of weeks for the SG and no-SG groups were 13.6 and 15.1, respectively. A Mann-Whitney *U* test indicated a significant difference between these two groups, *U*(*N*_*SG*_ = 57, *N*_*no-SG*_ = 89), 1911, *p* = .01, suggesting that a ‘dose-response’ relationship was not found and that participants who experienced a SG had on average a slightly shorter treatment duration. The baseline LSAS scores for the SG (*n* = 57, *M* = 85.74, *SD* = 21.35), and no-SG (*n* = 89, *M* = 80.12, *SD* = 18.88) groups were not significantly different, *p* = .10. Fig. 3 shows the symptom trajectories of the SG and non-SG groups.

### 3.3 SGs and reductions in social anxiety

A linear mixed-effects model was used to examine the differences in severity of social anxiety between participants with or without SGs. Pairwise comparisons showed that LSAS scores were lower among participants with SG than those without SG at post-intervention (*adjusted difference* = 25.22, SE = 3.07, *p* < .001, *d* = 1.23), and at three-month follow-up (*adjusted difference* = 20.78, SE = 3.15, *p* < .001, *d* = 1.01). Table 2 shows the summary statistics of the LSAS scores at post-intervention and follow-up.

**Table 2:** Means and Standard Deviations of unadjusted LSAS scores of the two groups.

	Mean (SD) at post-intervention	Mean (SD) at follow-up
Has SG	20.00 (13.24)	16.00 (12.00)
No SG	48.75 (26.06)	38.22 (25.97)

### 3.4 Process variables around the period of SGs

**Fig. 4:**
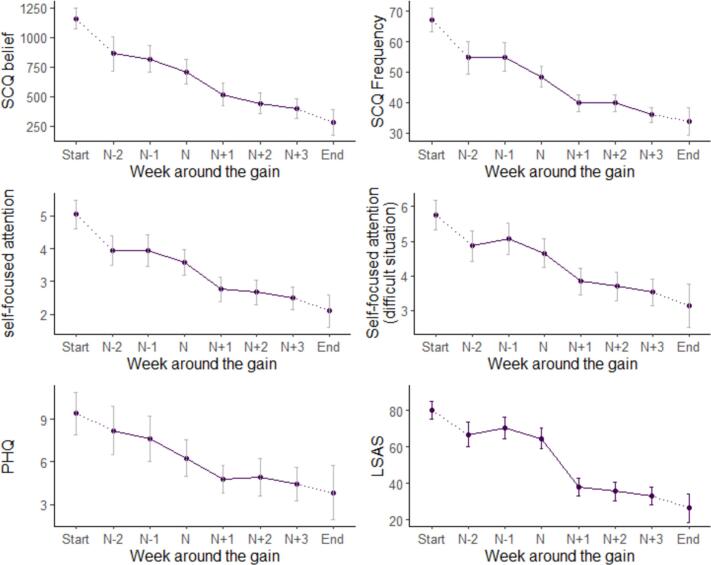
Social cognition, Self-focused attention, mood and social anxiety scores around the period of SGs.

Fig. 4 shows the scores on participants' process data around the period of SGs. The end of intervention was denoted as week 14 as this was the median end of treatment timepoint.

**Table 3:** Summary of Contrasts in Linear Mixed effects Models Examining Process Measures around the period of Sudden Gains.

Contrast	Estimate	Standard Error	p(Bonferroni-adjusted)	Cohen's *d*
SCQ Belief				
n-2 vs n-1	−106.33	42.95	0.146	0.53
n-1 vs n	−92.32	39.27	0.206	0.46
n vs n+1	−194.65	38.37	<0.001	0.96
n+1 vs n+2	−80.94	39.49	0.444	0.40
n+2 vs n+3	−30.43	40.62	1.000	0.15

SCQ Frequency				
n-2 vs n-1	−2.47	1.75	1.000	0.30
n-1 vs n	−5.81	1.60	0.003	0.70
n vs n+1	−8.64	1.56	<0.001	1.05
n+1 vs n+2	−0.34	1.61	1.000	0.04
n+2 vs n+3	−3.40	1.65	0.437	0.41

Self-focused attention (general)				
n-2 vs n-1	−0.20	0.23	1.000	0.19
n-1 vs n	−0.30	0.21	1.000	0.27
n vs n+1	−0.83	0.20	<0.001	0.76
n+1 vs n+2	−0.11	0.21	1.000	0.10
n+2 vs n+3	−0.13	0.22	1.000	0.12

Self-focused attention (difficult)				
n-2 vs n-1	0.13	0.25	1.000	0.11
n-1 vs n	−0.40	0.23	0.929	0.33
n vs n+1	−0.86	0.22	0.001	0.72
n+1 vs n+2	−0.12	0.23	1.000	0.10
n+2 vs n+3	−0.16	0.24	1.000	0.14

PHQ-9				
n-2 vs n-1	−0.63	0.68	1.000	0.20
n-1 vs n	−1.22	0.61	0.511	0.39
n vs n+1	−1.59	0.60	0.091	0.50
n+1 vs n+2	0.31	0.62	1.000	0.10
n+2 vs n+3	−0.59	0.64	1.000	0.18

LSAS				
n-2 vs n-1	0.30	1.82	1.000	0.03
n-1 vs n	−4.75	1.25	0.054	0.54
n vs n+1	−26.75	1.12	<0.001	3.03
n+1 vs n+2	−2.95	1.69	0.898	0.33
n+2 vs n+3	−1.71	1.69	1.000	0.19

Notes. SCQ = Social Cognitions Questionnaire; PHQ-9 = Patient Health Questionnaire; LSAS = Liebowitz Social Anxiety Scale.

Table 3 summarises the results of the linear mixed effects models. Bonferroni-adjusted pairwise linear contrasts indicated that all process variables except for mood significantly changed simultaneously with the gain (n to n+1). The frequency of negative social cognitions (SCQ frequency) also significantly declined in the interval prior to the SG (n-1 to n), but the LSAS score did not show a significant change across this interval (*p* = .054*).* Depressed mood did not significantly improve across any consecutive interval around the period of the gain. The unadjusted values of all measures around the period of the gain are shown in the supplementary material Table S1.

## 4 Discussion

The study examined the occurrence and frequency of sudden gains (SG) in routine clinical practice with a relatively large sample size (n = 146), and explored (1) the presence of SGs and its effect on treatment outcomes, and (2) the temporal relationship between changes in process variables (negative social cognition, self-focused attention and mood) and SGs. There was clear evidence for SGs, and process variables generally changed contemporaneously with improvement in social anxiety.

The proportion of participants experiencing one of more SGs was 39 % (57 participants out of 146 participants). This rate was comparable to the average SG occurrence rate of common mental health conditions (34.65 %) in a meta-analysis (Shalom and Aderka, 2020). The rates between Thew et al. (2023) (51 %) and the present study (39 %) were non-significant (*χ*^*2*^ = 2.16, *p* = .14).

There were a few differences between this study and the previous iCT-SAD study (Thew et al., 2023). The present therapists had less experience delivering the intervention. It is possible, for example, that they might have done less consolidation of key learning points during the phone calls, or other techniques that could have a bearing on whether SGs occur. The restrictions due to the COVID-19 pandemic might also play a role. Although one study on medical students with SAD found that the effect of CBT for SAD was sustained during the pandemic (Samantaray et al., 2022), COVID restrictions meant that social interactions were significantly reduced which limited opportunities for behavioural experiments. Participants might not have been able to experience a significant shift in their beliefs and anxiety by doing a single powerful experiment. However, these factors did not seem to affect the ocurrence of SG given the nonsignificance difference in SG rate.

Participants who experienced SGs had a significantly larger decrease in social anxiety from baseline to end-of-treatment and to follow-up. The effect sizes were large. The findings were similar to Thew et al. (2023) but not Hofmann et al. (2006), where they did not find an effect of SG on treatment outcome at follow-up. One of their speculations was the group format used in the study might have a moderating and stabilising effect on the trajectory of treatment gains. Hofmann et al. (2006) hence questioned the external validity of this phenomenon and wondered whether those were fluctuations in their symptoms. Nevertheless, the systematic review and meta-analysis by Shalom and Aderka (2020) found an overall predictive effect of SGs across common mental health conditions. The difference could potentially be accounted for by the difference in intervention used – Hofmann et al. (2006) examined a face-to-face group therapy and the intervention used in this study was an internet-based individual intervention.

This study demonstrated that cognitive and attentional changes co-occurred with the gain in a routine setting. Both the current study and Thew et al. (2023) showed a change in negative self-cognition preceeding the sudden gains, although the pattern of change was slightly different. Thew et al. (2023) showed a change in SCQ belief prior to the gain, and the present finding suggested the frequency of negative self-cognition was the main process of change prior to sudden gain. For mood, Thew et al. (2023) found it to change simultaneously with the gain, but it was not significantly different around the period of gain in the present study.

When looking at the distribution of pre-gain week (Fig. 2), weeks 2 had the highest occurrence of SGs. One might argue that early gains may be attributed to an expectation of improvement, or reengaging with past coping skills (Bisby et al., 2022). However, of note, the current intervention was intensive in the first two weeks (i.e. two phone calls with the therapist and completion of all the core modules), which might result in sudden gains earlier in therapy. In Week 2 in particular, participants complete a behavioural experiment targeting self-focused attention and safety-seeking behaviours which may lead to large cognitive changes.

Further efficacy and effectiveness studies are needed to ascertain the trajectory of change in these cognitive variables. In contrast, other studies such as Hofmann et al. (2006) did not find any evidence for cognitive changes preceding SG, and Bohn et al. (2013) found that cognitive changes did not precede SG but followed the gain which they suggested a possible ‘upward spiral’ effect (Tang and DeRubeis, 1999). One possible reason was the difference in sample size in the studies (*n* = 48 in the cognitive behavioural group therapy condition in Hofmann et al. (2006), *N* = 67 in Bohn et al. (2013)). Another possible reason was that the measure used to identify SGs were different – this study used the LSAS whereas Bohn et al. (2013) used the Social Phobia Weekly Summary Scale (SPWSS).

There is evidence to suggest that negative social cognitions and self-focused attention show substantial change across CT-SAD and that they mediate subsequent improvements in social anxiety symptoms (Thew et al., 2020). This would suggest that changes on these process variables around the period of a SG represent larger decreases within an overall decreasing trajectory. Future studies could helpfully examine the extent to which these process variable decreases differ between those who do and do not experience a SG.

### 4.1 Limitations and implications

There was no control group in the present routine setting, meaning we could not ascertain the extent of occurrence of SGs in the absence of treatment. Future studies could adopt a propensity-score case-control design. The measure of self-focused attention is a one-item measure, but it is widely adopted in studies on social anxiety. Reversal of SGs is an interesting concept to examine if it adversely impacts on recovery, but it is hard to draw firm conclusions as there were only three reversals in this study. Nevertheless, if this holds true in future studies, it may mean that iCT-SAD results in individuals having more stable improvement. Finally, although a temporal difference between change in SCQ frequency and LSAS score was demonstrated, the extent to which these are causally related is difficult to determine. This is because the LMMs do not account for potential unmeasured confounding variables that influence both SCQ frequency and LSAS scores. As an effectiveness study, we did not experimentally manipulate the variables to examine the differences. This is a common limitation for studies of SGs, and prospective experimental studies may be a helpful approach to overcome this. To increase the confidence in the causal relationship between cognitive processes and sudden gain, experimental paradigms such as dismantling RCT can be used in the future. Dismantling designs allow different components (e.g. cognitive and behavioural components) of the treatment to be compared with the combined intervention in separate research arms so that the unique effects of each component on sudden gains can be evaluated.

The present results are consistent with the view that SGs during iCT-SAD represent clinically meaningful events. Studies of individual patient trajectories have the potential to facilitate a better understanding of the mechanisms of change in psychological treatments for SAD. Further investigation of the specific therapeutic interventions occurring prior to SGs may help clarify potential mechanisms. It may also be useful to examine the differences between those experiencing sudden gains and those who experienced more gradual gains (i.e. people who achieved gains of a comparable size over non-consecutive sessions, see Greenfield et al., 2011). Clinically, the findings suggest it may be helpful for therapists to use routine outcome measures in order to track changes in process variables and observe when SGs occur.

To our knowledge, this study is the first to examine the occurrence of SGs during an internet intervention for SAD in a routine clinical care setting. The rate of SG was comparable to the average occurrence rate found in the systematic review (Shalom and Aderka, 2020). Sudden gains seem to be a common phenomenon across various therapy modalities and mental health conditions (Shalom and Aderka, 2020). It is perhaps possible there may be common processes leading to SGs across all modalities and disorders. However, in this study, SGs occurred at earlier points in therapy than other interventions. Thus it may be of clinical interest for future studies to compare how the modality, content, format and intensity of the intervention may impact on SGs and associated process variables. We found that reductions in negative social cognitions preceded SG and that changes in the degree of belief in these cognitions continued after the gain. Future studies could evaluate whether other processes, such as reductions in the use of safety behaviours, as described in the cognitive model, show a relationship with SG.

We did not collect qualitative data regarding SG in this study. Future studies could utilise qualitative methodologies to identify meaningful moments from the individuals' perspectives to help triangulate the findings. This may contribute to understanding the mechanisms of sudden gains examined by quantitative studies and what such ‘lightbulb moments’ mean to individuals. It would be meaningful to examine the magnitude of the cognitive or attentional change rather than simply examining the existence of change prior to the gain.

The following is the supplementary data related to this article.

Table S1 Unadjusted means and standard deviations of scores around the gain (n to n+1). Table S1

## Funding

This work was supported by the 10.13039/100010269Wellcome Trust [200796 (DMC)] and the National Institute for Health and Care Research (NIHR) Oxford Health Biomedical Research Centre. The views expressed are those of the authors and not necessarily those of the NIHR or the Department of Health and Social Care.

## Declaration of competing interest

The authors declare that they have no known competing financial interests or personal relationships that could have appeared to influence the work reported in this paper.
