# Supplementary material for: Sudden gains in internet cognitive therapy for social anxiety disorder in routine clinical practice
Source: Internet Interv. 2024 Nov 12;38:100788. doi: 10.1016/j.invent.2024.100788 (PMC11702001; doi:10.1016/j.invent.2024.100788)
Supplement: Table S1 — Unadjusted means and standard deviations of scores around the gain (n to n+1). [file mmc1.docx]

Supplementary Material

**Sudden Gains in internet cognitive therapy for social anxiety disorder in routine clinical practice**

Table S1. Unadjusted means and standard deviations of scores around the gain (n to n+1).

| **Timepoint** | **Mean** | **Standard Deviation** |
| --- | --- | --- |
|  | | |
| SCQ belief | | |
| n-2 | 861.86 | 484.15 |
| n-1 | 815.47 | 411.36 |
| n | 709.12 | 410.95 |
| n+1 | 516.84 | 366.41 |
| n+2 | 442.16 | 312.62 |
| n+3 | 394.81 | 299.36 |
| SCQ Frequency | | |
| n-2 | 54.72 | 17.99 |
| n-1 | 54.89 | 17.51 |
| n | 48.47 | 13.28 |
| n+1 | 39.91 | 10.70 |
| n+2 | 39.86 | 9.80 |
| n+3 | 35.94 | 9.06 |
| Self-focused attention (general) | | |
| n-2 | 3.95 | 1.50 |
| n-1 | 3.94 | 1.80 |
| n | 3.59 | 1.49 |
| n+1 | 2.76 | 1.45 |
| n+2 | 2.67 | 1.41 |
| n+3 | 2.50 | 1.26 |
| Self-focused attention (difficult) | | |
| n-2 | 4.86 | 1.49 |
| n-1 | 5.08 | 1.67 |
| n | 4.66 | 1.58 |
| n+1 | 3.84 | 1.50 |
| n+2 | 3.69 | 1.55 |
| n+3 | 3.52 | 1.38 |
| PHQ-9 | | |
| n-2 | 8.19 | 5.59 |
| n-1 | 7.62 | 5.87 |
| n | 6.25 | 4.95 |
| n+1 | 4.77 | 3.84 |
| n+2 | 4.92 | 4.74 |
| n+3 | 4.44 | 4.27 |
| LSAS | | |
| n-2 | 66.37 | 22.34 |
| n-1 | 70.19 | 21.28 |
| n | 64.22 | 22.39 |
| n+1 | 37.55 | 18.45 |
| n+2 | 35.57 | 19.13 |
| n+3 | 32.60 | 17.88 |

Notes. SCQ = Social Cognitions Questionnaire; PHQ-9 = Patient Health Questionnaire; LSAS = Liebowitz Social Anxiety Scale.
